# The role of body mass index in high- and low-velocity trauma causing knee injury associated with popliteal artery lesions

**DOI:** 10.1186/s13018-024-04821-w

**Published:** 2024-06-10

**Authors:** Andrea Ascoli Marchetti, Valerio Naldi, Vito Potenza, Fabio Massimo Oddi, Fernando De Maio, Riccardo Ciattaglia, Stefano Fazzini, Martina Battistini, Pasquale Farsetti, Arnaldo Ippoliti

**Affiliations:** 1 Unit of Vascular Surgery, Biomedicine and Prevention Department, University of Rome Tor Vergata, Viale Oxford 81, 00133 Rome, Italy; 2 Orthopedic and Traumatology Unit, Surgical Sciences Department, University of Rome Tor Vergata, Rome, Italy

**Keywords:** BMI, Knee trauma, Popliteal artery, Revascularization, Vein graft, Vascular injury, Multidisciplinary team

## Abstract

**Background:**

Among arterial traumas, osteoarticular traumas are particularly dangerous, and those involving the popliteal artery are associated with a high amputation rate. Despite representing a minority of arterial traumas, with an incidence that varies considerably by population and geographic location, traumatic lesions of the popliteal artery are challenging. This study aimed to verify the impact of body mass index (BMI) on arterial trauma damage and patient outcomes.

**Methods:**

Data were retrospectively collected from the electronic medical reports of all patients with osteoarticular and vascular associated lesions treated in the emergency operating room at our institution between 1 January 2005 and 1 May 2022. Forty-one patients presented with lower limb arterial trauma (43.2%); popliteal artery lesions occurred in 11 of these patients (26.8%), who were eligible for inclusion in the study. The lesion mechanism was dislocation by high-velocity trauma in 9 patients and dislocation by low-velocity trauma in 3 patients. All 7 males (63.6%) experienced high-velocity trauma, and 2 of the 3 females experienced low-velocity trauma. Only one patient had an isolated popliteal artery lesion associated with fractures in the leg or the contralateral limb. Patients with low-velocity trauma were older than 54 years, while those with high-velocity trauma were aged 22 to 71 years.

**Results:**

In 10/11 patients (90.9%), revascularization was performed after osteoarticular stabilization and reduction of the dislocation or fracture. Intraoperative angiography was selectively used. Two patients required above-the-knee amputation after the procedure: one due to infection of the surgical access point and the other due to severe soft tissue injury. One patient died during hospitalization due to trauma-related complications and comorbidities.

**Conclusions:**

High-velocity trauma and low-velocity trauma in patients with a body mass index > 35 kg/m^2^ and knee lesions are associated with popliteal artery lesions. Revascularization success is not associated with high- or low-velocity trauma.

## Background

**Fig. 1:**
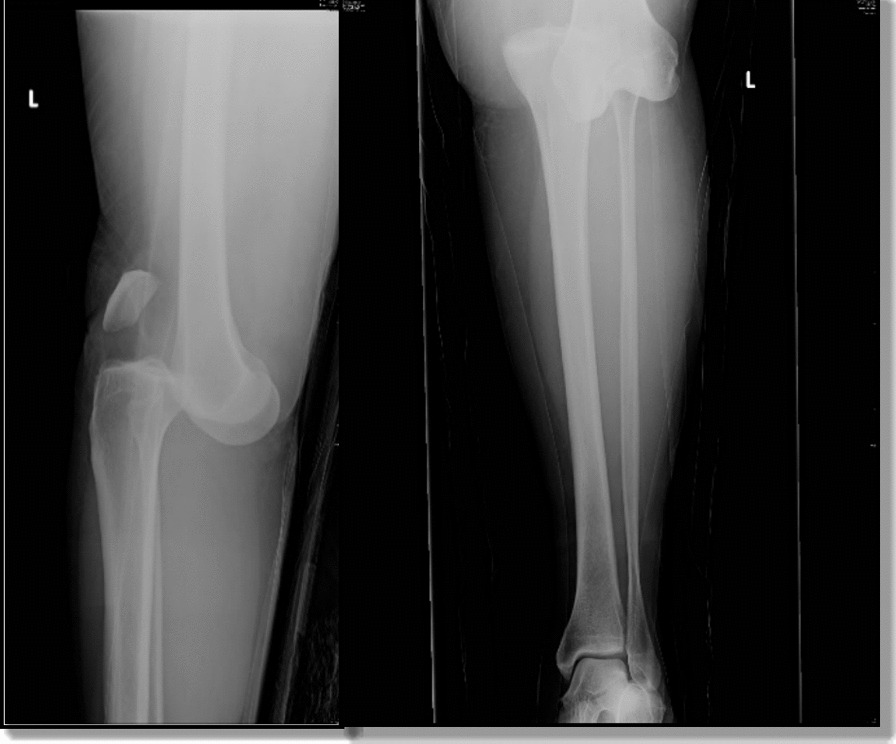
Patient 7. Plain RX: Posterior dislocation of the knee. Note the posterior descent of the bone segment of the femur

Traumatic lesions of the popliteal artery are rare [1, 2], with an incidence rate between 5 and 19% in the civilian population [3, 4]. Because of its anatomical position in the popliteal fossa, the popliteal artery is anteriorly protected by the knee joint and is rarely affected by isolated trauma. Damage to this artery is more frequently associated with knee fractures (Gustillo IIIC) 5,6 or knee dislocation, as in Patient 7 in our study (Fig. 1) [7, 8].

**Fig. 2:**
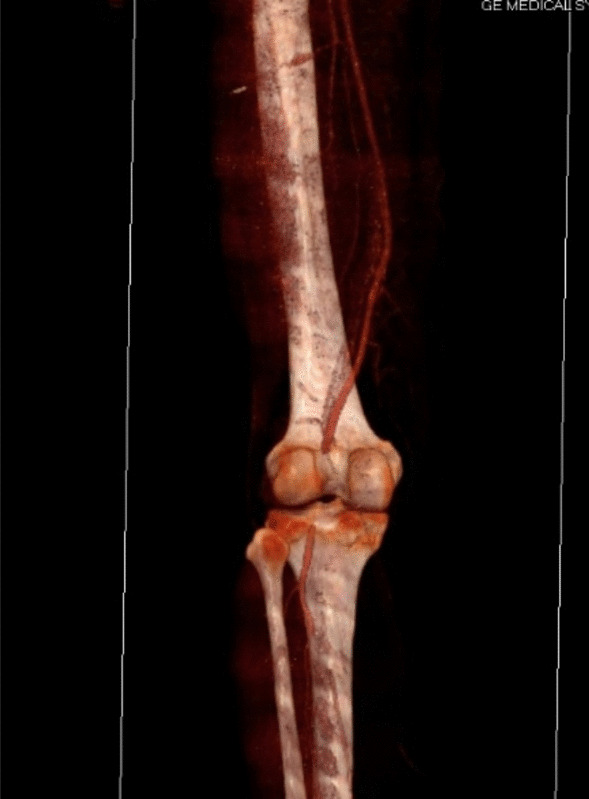
Patient 7, AngioCT scan of the lower left limb. After reduction of dislocation, an interruption of the passage of the contrast agent in the retroarticular popliteal artery was observed

Ligation of arterial injuries of the leg in World War II led to an amputation rate of 72% [9]; experience with arterial repair or reconstruction in the Korean War lowered the amputation rate to 32% [10]. More than a decade later, similar amputation rates were reported during the Vietnam War [11]. Although a much lower amputation rate was reported between 1990 and 2000, traumatic lesions of the popliteal artery continue to be the most common arterial lesion associated with limb loss [12, 13]. A multidisciplinary approach is key in the successful management of knee injury. Dislocation or fracture can cause a vascular lesion, which must be recognized quickly to achieve imminent repair. The osteoarticular cause must also be identified for appropriate revascularization. Surgeons should always consider the possibility of a popliteal artery lesion, even in cases of minor trauma and after reduction of a dislocation [14, 15] (Fig. 2).

Few studies have focused on the relationship between speed of the trauma and body mass. This study aimed to evaluate risk factors of trauma associated with arterial injury and verify the role of body mass in the outcome of surgical arterial revascularization.

## Methods

This was a retrospective analysis from a single centre. No ethical approval or relevant judgement from ethics committees was needed. Prior to beginning the writing of this article, all patients and/or family members were contacted to seek their consent for the release and processing of sensitive data for research purposes. The main inclusion criteria were traumatic lesions of the popliteal artery and age older than 18 years at the time of admission. The exclusion criteria were arterial lesions not involving the limbs and the absence of a diagnosed popliteal artery lesion (such as popliteal artery aneurysm) prior to trauma and admission to the hospital. The patients were divided into two study groups based on the dynamics of the trauma that caused the arterial lesion: high or low velocity. A high-velocity trauma was defined as an incident involving a motorized vehicle, such as motor vehicle collisions, motorcycle collisions, moped collisions, motorized vehicle collisions, or crush injuries. A low-velocity trauma was defined as that occurring secondary to a fall, sport, or assault [8, 14].

Clinical and operative data were retrospectively collected from the electronic medical reports of emergency and operating rooms at Tor Vergata Hospital between 1 January 2005 and 1 May 2022. In that time, ninety-five patients were admitted to Tor Vergata Hospital with a diagnosis of arterial trauma (Table 1).

**Table 1:**
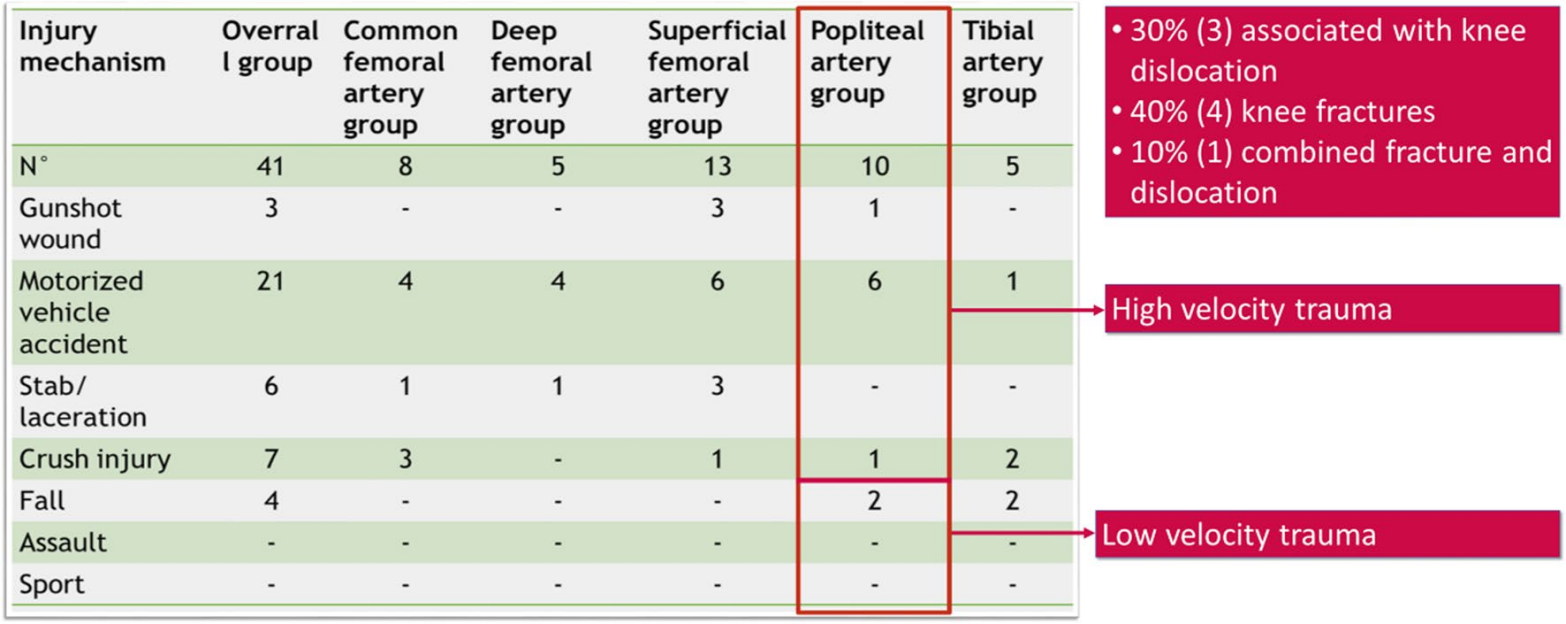
Patients admitted and mechanism of trauma with arterial involvement

The inclusion criterion was popliteal artery injury; 42 patients presented with lower limb arterial trauma (44.2%), and of these patients, popliteal artery lesions occurred in 11 (26.8%) patients. Ten patients were eligible for inclusion in the study. The lesion mechanism was dislocation by high-velocity trauma in 9 patients and dislocation by low-velocity trauma in 2 patients. All 7 males (70%) experienced high-velocity trauma, and 2 of the 3 females experienced low-velocity trauma. Three patients had popliteal artery lesions and knee dislocation (all posterior dislocations), two of whom had a total knee prosthesis; five patients had knee fractures associated with popliteal artery trauma; and one patient had knee fracture and dislocation involving the popliteal artery. Only one patient had an isolated popliteal artery lesion associated with fractures in the leg or the contralateral limb. Patients with low-velocity trauma were older than 54 years, while those with high-velocity trauma were aged 22 to 71 years.

## Statistical analysis

The data was extracted from the electronic health record system. The intended proforma was completed which included age, sex, weight as baseline demographic data. Surgical data including surgery site, year of operation, and primary patency of the arterial substitute. Postoperative early (hematoma, infection, seroma) and late (thrombosis, stenosis) complications and their management were also documented A minimum of 6 and a maximum of 24 months of follow-up was considered. After 6, 12, 18, and 24 months, the primary, primary assisted, and secondary patencies were calculated using these data.

In SPSS version 20, all the data were entered and examined. Age, associated lesions, patency, and follow-up duration are all continuous variables that were provided as mean and standard deviation. Sex, surgery site, early and late problems, and complications are categorical variables that were provided as frequency and percentages. The indications for open surgical repair and the open surgical approach and technique used were recorded and analysed. Additionally, complications after open surgery, intraoperative death, length of stay, hospital mortality, long-term mortality, and reintervention rates were assessed. Groups were compared with nonparametric statistical tests; categorical variables were compared.

## Results

**Fig. 3:**
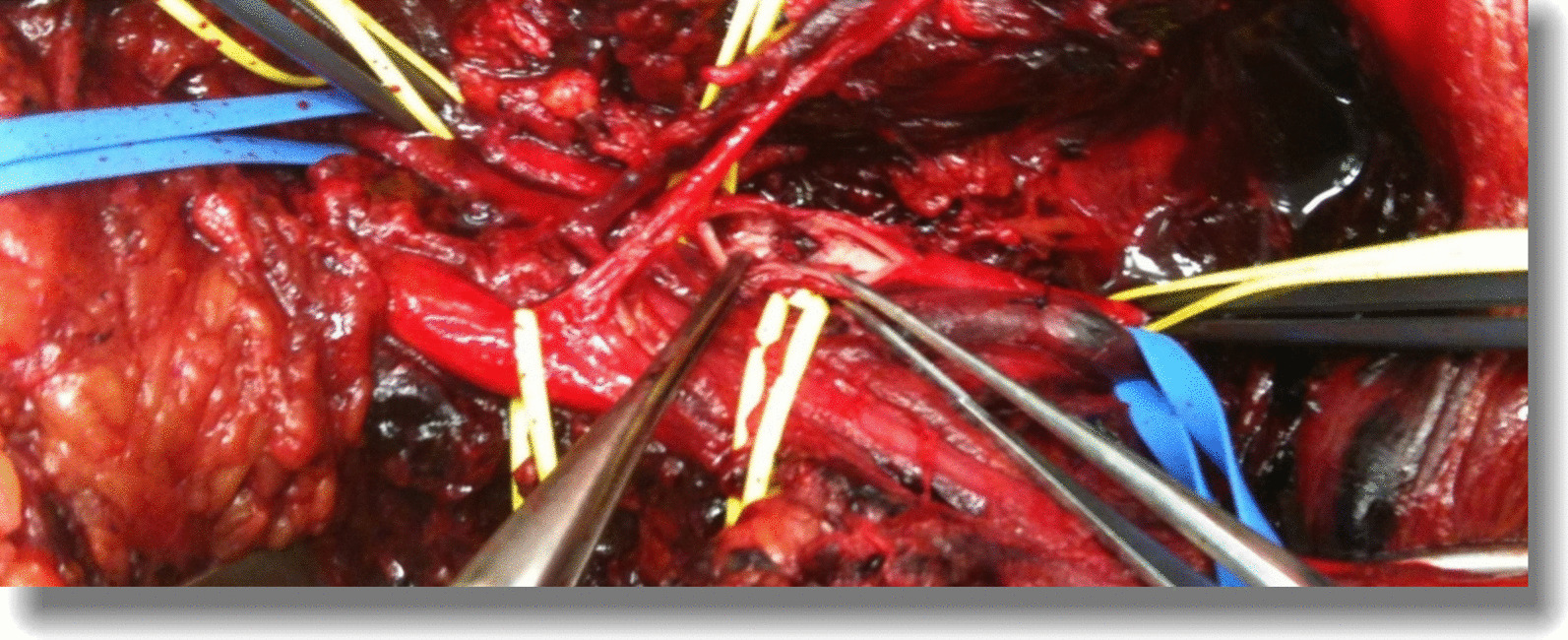
Patient 7, intraoperative image. Posterior popliteal surgical access with popliteal artery clamping and longitudinal arteriotomy After removal of the intraluminal clot, the interruption of the tunica intima, the seat of the arterial trauma, was observed

All patients were treated within 3 h from admission to the emergency room and within 6 h from the accident. In eight patients, revascularization was performed after osteoarticular stabilization, except for one patient in whom revascularization was performed before orthopaedic surgery due to bleeding from the artery. One patient required arterial reconstruction without subsequent collaboration with orthopaedists due to the absence of fractures or dislocations. Posterior surgical access to the knee was used in most patients with popliteal area involvement (9/11 (81,8%)) (Fig. 3), with only two patients (18.1%) requiring the medial approach.

The posterior approach was chosen to allow orthopaedists to work with a single access point, but patients with particularly complicated situations, especially posterior knee dislocations or pluriframmentary fractures, required the medial approach. During every surgery, systemic heparinization was performed, except for one haemophilic patient with an increased risk of intraoperative bleeding. Treatments were performed depending on the lesion and the availability of a great saphenous vein (GSV) of adequate calibre (at least 3 mm in diameter); where possible, end-to-end anastomosis of the popliteal artery was preferred. Four patients were treated with GSV grafts, four with end-to-end anastomosis of the popliteal artery without any grafts, and three with synthetic grafting due to an inadequate GSV calibre. Revascularization of the popliteal artery was successful in seven out of 11 patients, two of whom underwent amputation above the knee. One of the two patients was a 54-year-old obese woman (BMI = 40 kg/m^2^) who had rheumatoid arthritis and drug-induced osteoporosis due to corticosteroid therapy and who experienced a low-velocity trauma from falling. Her knee prosthesis was dislocated after her fall from an upright position, which injured her popliteal artery. Because of her immune dysfunction due to rheumatoid arthritis and because she was administered corticosteroid therapy, she contracted a multidrug-resistant infection of the surgical wound that made amputation necessary after 2 months of medical therapy. The second patient was a 24-year-old man who attempted suicide by falling from a height of 12 m; he underwent bilateral GSV graft revascularization and osteoarticular stabilization but ultimately required above-the-knee amputation due to extensive soft tissue injury. A third patient whose condition was worsened due to haemophilia type A died 6 months after the GSV procedure due to the extensive injuries caused by the accident. Despite prolonged antibiotic therapy, septic shock combined with traumatic soft tissue injuries resulted in the patient's death, but graft patency was maintained. All patients were intraoperatively subjected to Eco-duplex. Intraoperative angiography was performed in only one patient. The patients treated with revascularization due to popliteal artery injury are described in Table 2, which shows that there was no difference in revascularization success between high-velocity and low-velocity trauma patients due to the small number of cases reported.

**Table 2:** Demographic data, mechanism of injury and postoperative course of trauma

Pt	Sex	Age	BMI(kg/m^2^)	Trauma dynamic	Osteoarticular trauma	Revascularization	Postoperative course	Comorbidity
1	F	54	40,2	Low speed	Dislocation of the knee prosthesis	End-to-end anastomosis	Amputation above the knee	Rheumatoid arthritis, osteoporosis, total knee prosthesis, hip prosthesis (bilateral)
2	M	35	27	High speed	Proximal tibial epiphysis fracture	GSV graft	Discharged,maintaining patency	–
3	M	65	31,5	High speed	Dislocation of the knee prosthesis	ePTFE synthetic graft	Discharged,maintaining patency	Arthrosis, total knee prosthesis
4	M	52	25	High speed	Distal femoral epiphysis fracture, proximal tibial epiphysis	ePTFE synthetic graft	Discharged,maintaining patency	–
5	M	57	18,7	High speed	Knee dislocation, vertebral L1 fracture, pelvis fracture	GSV graft	Deceased due to traumatic soft tissue lesions, infection and sepsis	Haemophilia A
6	M	22	26,6	High speed	Proximal tibial epiphysis fracture, tibial diaphysis fracture, fibula fracture	End-to-end anastomosis	Discharged,maintaining patency	–
7	F	57	35,8	Low speed	Knee dislocation	GSV graft	Discharged,maintaining patency	
8	M*	24	25	High speed	Proximal tibial epiphysis fracture, distal femoral epiphysis fracture, tibial multifragmentary fracture	GSV graft	Amputation above the knee due to extensive soft tissue lesions	
9	M*	24	25	High speed	Distal femoral epiphysis fracture, tibial multifragmentary fracture	GSV graft	Discharged,maintaining patency	
10	F	71	23	High speed	Proximal tibial epiphysis fracture	End-to-end anastomosis	Discharged,maintaining patency	Arthrosis, osteoporosis
11	M	37	28,3	High speed	Colles fracture	ePTFE synthetic graft	–	–

One patient died during hospitalization due to trauma-related complications and comorbidities, namely, haemophilia type A, despite maintaining graft patency. Among the complications, two patients had deep venous thromboses (6.4%), and two patients (6.4%) healed by secondary intention. Follow-up data were available for 6 of the 8 salvaged limbs over a mean 12-month period, during which patency of arterial reconstruction, vein graft, or synthetic graft was maintained in all patients. Furthermore, 1-year patency was maintained in three patients with end-to-end anastomoses, one patient with a GSV graft, and two patients with synthetic grafts (Tables 3 and 4).

**Table 3:** Preoperative and intraoperative data

Time interval between trauma and intervention hours	Operative timing: Prior orthop vs vascular N° cases	Posterior Surgical approach N° cases	Material for arterial repair Vein Sintetic. End-end		
3 (min 1,5-max 6)	10/11 (90,9%)	9/11 (81.8%)	4 /11(36.3%)	4 /11(36.3%)	3/11 27.2%)

**Table 4:** Postoperative results and complication

Immediate patency	Postoperative DVT	Healing by secondary intention	Amputation rate
11/11 (100%)	2/11 (18.1%)	2/11 (18.1%)	2/11 (18.1%)*

^*^With patent artery

## Discussion

Popliteal artery traumas represent a minority of arterial traumas, likely due to their anatomical position in the popliteal fossa and posterior to the knee joint. Unless there are preexisting structural lesions (aneurysms) or genetic predispositions such as collagenopathy or Ehlers–Danlos syndrome (laxity of joint ligaments), high-velocity trauma is required to disrupt the knee joint. Although rare, cases of low-velocity trauma exist; these can cause damage to the joint and involve the popliteal artery. This is dangerous because the low velocity of trauma associated with a large weight can be equally dangerous, and signs and symptoms may be inconsistent; typical signs of arterial injury are absent in up to 40% of patients, where the presence of a pulse in the affected limb does not exclude an arterial lesion and segmental Doppler may not detect the injury [16–19]. This type of injury does not depend only on the trauma dynamic (as with high-velocity traumas) but also on the patient’s risk factors. According to the literature, the keys for effective revascularization are systemic heparinization [20–22], revascularization time between 5 and 8 h after trauma, and end-to-end anastomosis instead of using a GSV graft or synthetic graft. Wherever possible, we preferred end-to-end anastomosis over GSV grafting because anastomosis is associated with a higher success rate [13]. Furthermore, several authors have suggested that fibromuscular tethering of the artery in the popliteal fossa precludes tension-free anastomosis unless potentially critical perigeniculate collaterals are divided. Therefore, interposition or bypass grafting is advocated in these cases [20, 23, 24]. The extension of soft tissue injuries is crucial for adequate and successful revascularization and explain why amputation rates due to arterial injury from secondary blunt trauma are twice as great as those due to injury from penetrating trauma [25]. Patients affected by low-velocity trauma had a BMI greater than 35 kg/m^2^, while patients affected by high-velocity trauma had a BMI between 18.7 and 31.5 kg/m^2^. The advanced age of patients affected by low-velocity trauma could represent a predisposing factor with regard to diseases associated with ageing, such as osteoporosis, arthrosis, and ligamentous laxity, rendering them prone to fractures or dislocations, including of the knee joint, as body weight represents a strong risk factor for low-velocity trauma fractures and dislocations [8, 14, 26]. Three of our patients had knee prosthesis dislocation, a rare but possible cause of popliteal artery injury, especially posterior dislocation; under these conditions, the disruption of ligaments was also described by Bonnevialle et al. in concomitant palsy of the common peroneal nerve [27, 28]. Our findings that there was no correlation between the dynamics of the trauma and the success of revascularization are consistent with those in the literature. This study has several limitations. The first limitation was the small number of patients since our institution is not a trauma centre, and popliteal artery trauma has a low incidence. Furthermore, according to the literature reviews, there is no single definition of low- or high-velocity trauma. Therefore, different articles may have different results depending on their interpretations. Finally, as the patients in this study were from a specific population, the findings about mass/velocity data cannot be extrapolated to the general population. Statistically significant conclusions can be drawn only after additional data are collected. However, the correlation found in this study remains suggestive and can be explored in more depth in further studies.

## Conclusion

Lesions of the popliteal artery following low-velocity trauma are correlated with a high BMI > 35 kg/m^2^ and must be considered in orthopaedic evaluation. In addition, trauma dynamics (low- or high-velocity) do not influence the success of revascularization. Instead, factors that influence the results include the revascularization procedure occurring between 6 and 8 h after the accident, intraoperative systemic heparinization, and an appropriate technique for revascularization and extension of soft tissue injuries. There may be an association between age and susceptibility to arterial low-velocity trauma; however, many additional risk factors should be analysed, and a greater number of cases are needed. Further in-depth studies should be conducted to support this association.

## Data Availability

All clinical and operative data were available in the electronic medical reports of the emergency and operating rooms at our institution.

## Abbreviations

BMI: Body mass index
GSV: Great saphenous vein
